# Reply to Legat, B.; Rocher, L. The Limits of Pairwise Correlation to Model the Joint Entropy. Comment on “Nguyen Thi Thanh et al. Entropy Correlation and Its Impacts on Data Aggregation in a Wireless Sensor Network. *Sensors* 2018, *18*, 3118”

**DOI:** 10.3390/s21113729

**Published:** 2021-05-27

**Authors:** Nga Nguyen Thi Thanh, Khanh Nguyen Kim, Son Ngo Hong, Trung Ngo Lam

**Affiliations:** School of Information and Communication Technology, Hanoi University of Science and Technology, Hanoi 11615, Vietnam; khanhnk@soict.hust.edu.vn (K.N.K.); sonnh@soict.hust.edu.vn (S.N.H.); trungnl@soict.hust.edu.vn (T.N.L.)

In the comment, the authors have mentioned that two claims in our paper are incorrect in general. On behalf of all authors, I would like to reply as follows:

- As mentioned in our paper, claim 1 derives from [1,2] such that the correlation coefficient between one cluster and another cluster can be obtained by the smallest/largest/average correlation coefficient from any member of one cluster to any member of the other. Claim 2 is proved by using claim 1.
- We believe that there is a class of datasets such as environmental parameters (temperature as shown in our paper) and vision data (as shown in [1]) that satisfy claim 1. The next work is to find the properties of these datasets and clarify the application range of our result.
